# Influenza‐associated mortality in South Africa, 2009‐2013: The importance of choices related to influenza infection proxies

**DOI:** 10.1111/irv.12498

**Published:** 2017-12-02

**Authors:** Desmond Gul, Cheryl Cohen, Stefano Tempia, Anthony T. Newall, David J. Muscatello

**Affiliations:** ^1^ School of Public Health and Community Medicine University of New South Wales Sydney NSW Australia; ^2^ Center for Respiratory Diseases and Meningitis National Institute for Communicable Diseases of the National Health Laboratory Service Johannesburg South Africa; ^3^ Influenza Division Centers for Disease Control and Prevention Atlanta GA USA; ^4^ Influenza Program Centers for Disease Control and Prevention Pretoria South Africa

**Keywords:** influenza, mortality, proxy, South Africa, time‐series study

## Abstract

**Background:**

Regression modeling methods are commonly used to estimate influenza‐associated mortality using covariates such as laboratory‐confirmed influenza activity in the population as a proxy of influenza incidence.

**Objective:**

We examined the choices of influenza proxies that can be used from influenza laboratory surveillance data and their impact on influenza‐associated mortality estimates.

**Method:**

Semiparametric generalized additive models with a smoothing spline were applied on national mortality data from South Africa and influenza surveillance data as covariates to obtain influenza‐associated mortality estimates from respiratory causes from 2009 to 2013. Proxies examined included alternative ways of expressing influenza laboratory surveillance data such as weekly or yearly proportion or rate of positive samples, using influenza subtypes, or total influenza data and expressing the data as influenza season‐specific or across all seasons.

**Result:**

Based on model fit, weekly proportion and influenza subtype‐specific proxy formulation provided the best fit. The choice of proxies used gave large differences to mortality estimates, but the 95% confidence interval of these estimates overlaps.

**Conclusion:**

Regardless of proxy chosen, mortality estimates produced may be broadly consistent and not statistically significant for public health practice.

## 1 INTRODUCTION

Estimation of the mortality burden of seasonal and pandemic influenza is important in public health as it can be used to inform the impact of influenza control policies and programs; however, such estimates are not easy to ascertain. Using influenza‐coded deaths usually grossly underestimate the burden of influenza‐associated deaths,^1^ as these deaths are more often complicated by secondary bacterial co‐infections or exacerbation of underlying chronic conditions^2^ or even cardiac complications.^3^ These deaths are usually recorded with an underlying cause of death other than influenza. In South Africa, the mortality risk is further compounded by high HIV prevalence, which puts HIV patients at a much higher risk of influenza‐related mortality and other opportunistic infections.^4^ This adds another level of uncertainty in the recorded underlying cause of death.

To overcome underestimation of influenza‐related deaths, ecological time‐series modeling is used to estimate influenza's association with a broader range of coded causes of death. Many of these methods originate from the original Serfling regression approach,^5^ with numerous adaptations.^6^, ^7^ These approaches are based on the long‐recognized elevation in weekly or monthly time series of deaths that occurs when influenza is circulating in populations. The population rate of influenza‐associated deaths is obtained by estimating excess mortality by subtracting a seasonally varying background rate of non‐influenza deaths from observed death rates.^5^ A more convincing modeling approach includes independent covariates representing trends in incidence of influenza infection in the model. Influenza‐associated excess mortality can then be estimated using the parameter estimates of the influenza variables of the model, which represent the relationship between observed trends in influenza and the modeled mortality outcomes. Indicators or proxies for the incidence of influenza infection include time series of laboratory‐confirmed influenza infections in influenza‐like illness (ILI) surveillance from sentinel health facilities.^8^, ^9^, ^10^

Laboratory surveillance time series can be expressed in various ways. Firstly, proxies could be expressed as weekly or monthly counts of total influenza‐positive samples or proportion positive of samples tested. Using proportions standardizes the data, automatically adjusting for changes in testing frequency and laboratory procedures over time.^11^, ^12^ On the other hand, the proportion may be influenced by factors other than influenza—the denominator may vary due to the incidence of non‐influenza causes of acute respiratory infection.

Proxies can be further disaggregated by influenza virus types (A and B) and subtypes of influenza A, such as A(H3N2) and A(H1N1)pdm09, if laboratory surveillance data are sufficiently refined. While several influenza types and subtypes (henceforth denoted as (sub)types) cocirculate, relative timing and incidence can vary.^8^ In addition, virus virulence can vary from season to season, even within the same type or subtype.^13^ To account for varying virulence, a separate influenza‐incidence proxy can be used for each season.^14^

It is unknown which proxy provides the best estimates in ecological time‐series studies. Several studies estimating influenza‐associated mortality in South Africa used monthly proportions of total influenza‐positive viral samples, with the denominator being the total annual number of samples tested.^15^, ^16^, ^17^ The aim of this study is to look at how estimated influenza‐associated mortality rates are affected by the choice of influenza proxies used.

## 2 METHODS

### 2.1 Study period and setting

The study period included 260 weeks (Monday–Sunday) from 5 January 2009 to 29 December 2013 inclusive. The setting was the nation of South Africa with a population of about 53 million in 2013.

### 2.2 Mortality data and population denominators

Non‐identified data on underlying causes of death from 2009 to 2013 for the South Africa population are publicly available.^18^ Causes of death are coded using the International Classification of Diseases, Tenth Revision (ICD‐10). Weekly time series of mortality counts for respiratory death (ICD‐10: J00‐J99) were prepared for persons of all ages, aged <65 and ≥65 years. Records with missing age (0.27% of total deaths) were excluded.

Population denominators for rates were obtained from mid‐year population estimates from Statistics South Africa^19^ and were linearly interpolated to provide denominators for weekly death rates.

### 2.3 Influenza surveillance data and proxies

Information on circulating influenza viruses was obtained from the South African Severe Acute Respiratory Illness (SARI) surveillance program, which is a prospective, hospital‐based sentinel surveillance program that covers 4 of 9 provinces in South Africa.^20^ Respiratory samples were tested by real‐time reverse‐transcription polymerase chain reaction, and influenza viruses identified were typed as influenza A or B. Influenza A viruses were subtyped into influenza A(H1N1)pdm09 or influenza A(H3N2).

The 3 influenza‐incidence proxy variables we evaluated are summarized in Table 1. First, weekly population rates of influenza‐positive specimens were used to account for changing population size. The other 2 methods were calculated as proportions of positive specimens. The “weekly proportion” was simply the weekly number of specimens testing positive for each influenza (sub)type by the total number of specimens tested for that week. The “annual proportion” was calculated by dividing the weekly number of specimens testing positive for each (sub)type by the total number of specimens tested in the year of analysis.

**Table 1:** Definitions of influenza proxies compared in models to estimate influenza‐associated respiratory deaths

Model	Definition of influenza‐incidence proxy						
	All‐season^a^	Season‐specific^b^	All‐influenza^c^	Influenza (sub)types^d^	Weekly proportion^e^	Yearly proportion^f^	Rate^g^
1	✓		✓		✓		
2	✓		✓			✓	
3	✓		✓				✓
4	✓			✓	✓		
5	✓			✓		✓	
6	✓			✓			✓
7		✓	✓		✓		
8		✓	✓			✓	
9		✓	✓				✓
10		✓		✓	✓		
11		✓		✓		✓	
12		✓		✓			✓

a All‐season: influenza proxy for whole study period is analyzed as a whole.

b Season‐specific: influenza proxy for each season analyzed as individual covariates.

c All‐influenza: influenza proxy of weekly positive samples is not subtyped but considered as a whole.

d Influenza (sub)types: proxy consisting of influenza types and subtypes analyzed as individuals covariates—A(H1N1)pdm09, A(H3N2), and B.

e Weekly proportion: proxy calculated as total weekly samples testing positive for influenza viruses divided by total weekly samples tested.

f Yearly proportion: proxy calculated as total weekly samples testing positive for influenza viruses divided by total yearly samples tested.

g Rate: proxy calculated as total weekly samples testing positive for influenza viruses divided by weekly population in South Africa.

We also looked at allowing the relationship between viral proxies and influenza‐associated mortality to vary by year, to accommodate annual changes in virus virulence or changes in the surveillance program. Each influenza (sub)type in a particular season therefore is represented by an individual covariate, which is set to 0 in all other seasons.^14^ Proxy variables for these were termed “season‐specific” as opposed to “all‐season,” which considered a single viral proxy variable for each virus category across all seasons.

### 2.4 Statistical analysis

Statistical analysis was conducted using the generalized additive model (GAM) procedure in SAS 9.3 (SAS Institute Inc., Cary, NC, USA). Weekly rates of influenza‐associated deaths from respiratory causes were estimated using a semiparametric GAM. Independent variables of influenza‐incidence proxies were parametrically and linearly related to respiratory mortality incidence. We used a natural cubic smoothing spline of weeks to account for the time trend in non‐influenza‐associated (background) mortality. In an Australian study, the GAM model formulation provided an improved model fit compared with the more conventional trigonometric (sinusoidal) background mortality estimation approach.^21^

The final model equations for each of the proxy definitions above are as follows:

1. Influenza (sub)type, all‐season formulation: $\begin{array}{cc} E(\text{mortality}\;\text{rate})\;=\; & \beta_{0}\;+\;\beta_{1}t+\beta_{2}\left(\text{Influenza}\;\text{A(H1N1)}\right)\\ & +\beta_{3}\left(\text{Influenza}\;\text{A(H3N2}\right))+\beta_{4}\left(\text{Influenza}\;B\right)+\text{spline}\left(t\right) \end{array}$
2. All‐influenza, all‐season formulation: $E\left(\text{mortality}\;\text{rate}\right)=\beta_{0}+\beta_{1}t+\beta_{2}\left(\text{all‐}\;\text{influenza}\right)+\text{spline}\left(t\right)$
3. Influenza (sub)type, season‐specific formulation: $\begin{array}{cc} E(\text{mortality}\;\text{rate})\;=\; & \beta_{0}+\beta_{1}t+[\sum_{\left(y=2009\right)}^{2013}\beta_{2,y}\left(\text{Influenza}\;\text{A(H1N1)}\right)]\\ & +[\sum_{\left(y\;=\;2009\right)}^{2013}\beta_{3,y}(\text{Influenza}\;\text{A(H3N2)}]\\ & +[\sum_{\left(y\;=\;2009\right)}^{2013}\beta_{4,y}\left(\text{Influenza}\;B\right)]+\text{spline}\left(t\right) \end{array}$
4. All‐influenza, all‐season formulation: $E\left(\text{mortality}\;\text{rate}\right)=\beta_{0}+\beta_{1}t+[\sum_{\left(y\;=\;2009\right)}^{2013}\beta_{2,y}\left(\text{all}\text{‐}\;\text{influenza}\right)]+\text{spline}\left(t\right),$

where *E*(mortality rate) was the expected respiratory mortality rate, *t* was the sequential week number of the weekly time series, Influenza A(H1N1) was the proxy for influenza A(H1N1)pdm09, Influenza A(H3N2) was the proxy for influenza A(H3N2), Influenza B was the proxy for influenza B, all‐influenza was the aggregated proxy for all 3 influenza viruses, and spline was the spline curve for *t* and was specified with 31 degrees of freedom which achieved a degree of control for autocorrelation (*r* < .2). One degree of freedom is allocated to the parametric linear time variable (β_1_ *t*), and the remaining degrees of freedom are distributed at 6 per year for the spline.^21^

Estimated background mortality was calculated from the model by setting all the influenza variables to zero and introducing them into the fitted model formula. The estimated influenza‐associated excess death rate in each week was determined by multiplying the influenza parameter estimate (β) by its respective influenza surveillance proxy value. Negative influenza parameter estimates led to negative mortality estimates, which are biologically meaningless,^14^ and were regarded as zero when aggregating estimates for (sub)type‐specific influenza. The 95% confidence interval (CI) of the estimated mortality rate was obtained by multiplying the influenza surveillance proxy value using each of the upper and lower 95% CI as shown below:

β±(1.96×SE),

where SE is the standard error of the parameter estimate, β.

The standard error for the 95% CI for aggregate (sub)type‐specific estimates was calculated as:

$$\text{SE}=\sqrt{{\text{SE}}_{1}^{2}+\cdots +{\text{SE}}_{n}^{2}},$$

where SE_1_…SE_n_ are standard errors of aggregated proxies used.

An equivalent procedure was used to estimate the 95% CI for the annual mean influenza‐attributable mortality estimates.

Model fit was assessed by the square root of the mean‐squared error (RMSE) of the model over the entire time series. A lower value indicates improved fit.^22^

### 2.5 Ethics

The study was approved by the Human Research Ethics Committee of the University of New South Wales, Australia. The SARI protocol was approved by the University of the Witwatersrand Human Research Ethics Committee (HREC) and the University of KwaZulu‐Natal Human Biomedical Research Ethics Committee (BREC) protocol numbers M081042 and BF157/08, respectively. This surveillance was deemed non‐research by the US Centers for Disease Control and Prevention.

## 3 RESULTS

### 3.1 Influenza surveillance

**Figure 1:**
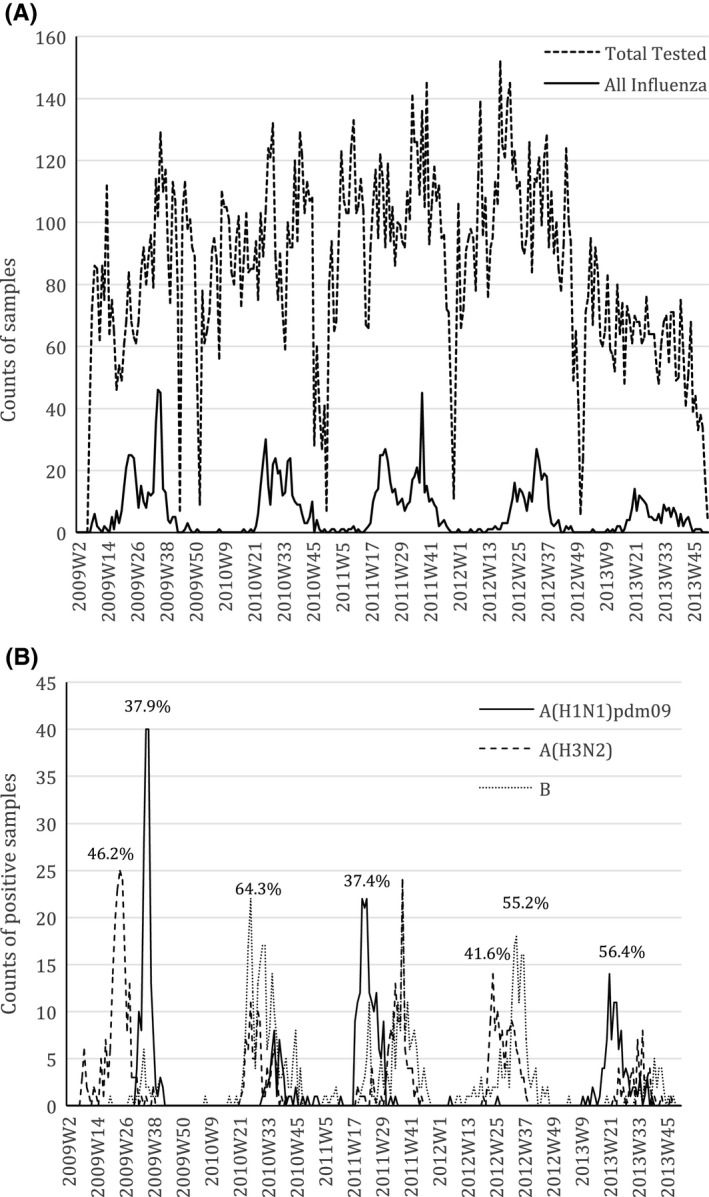
Influenza surveillance time series of count of influenza‐positive samples tested in South Africa from 2009 to 2013. A, All‐influenza‐positive samples and total samples tested; B, individual influenza type‐ or subtype‐positive samples. Numbers above peaks in (B) represent percentage of dominant influenza type or subtype in circulation for that year. The *x*‐axis depicts the week during the study period, indicated firstly by year followed by the week number, W1‐W52, in that year

A mean of 4330 respiratory specimens were tested annually for influenza viruses. The mean annual number of specimens that tested positive for one or more influenza viruses was 332 (7.7%). Over the study period, different influenza (sub)types circulated at varying times throughout the year and their relative annual proportions are shown in Figure 1. Time series of influenza surveillance as weekly proportion, yearly proportion, and population rate is shown in Figure S1. In 2009, influenza virus activity occurred in 2 peaks; influenza A(H3N2) dominated from May to July, and a second peak occurred from August to September with the introduction of influenza A(H1N1)pdm09.

### 3.2 Observed mortality rate

During the study period, 302 112 respiratory deaths were recorded. The mean respiratory mortality weekly rate over the study period was 2.29 per 100 000 population for all ages, 1.69 per 100 000 population for persons aged <65 years, and 13.49 per 100 000 population for persons aged ≥65 years. A marked downward trend in mortality rate can be seen in the all age and <65 years age groups (Figure S2).

### 3.3 Goodness‐of‐fit model

The best model fit as assessed by the lowest RMSE value in both age groups and all ages combined was obtained by using weekly proportion of influenza (sub)types as proxies (Table 2). Best fit was not confined to all‐season or season‐specific proxies. In the all‐age group, the use of season‐specific proxy variables gave better fit than all‐season proxy variables. In the 2 age‐stratified groups, all‐season proxy variables improved fit.

**Table 2:** Assessing goodness‐of‐fit models by root of the mean‐squared error (RMSE) values on choice of proxies used, age‐stratified, in South Africa, 2009‐2013

Age group	RMSE^a^	Model proxy characteristics		
		Proxy calculation	Subtype or all‐influenza	Season‐specific or all‐season
All ages	.1121	Weekly proportion	Subtype	Season‐specific
	.1143	Yearly proportion	Subtype	Season‐specific
	.1143	Rate	Subtype	Season‐specific
	.1166	Weekly proportion	Subtype	All‐season
	.1181	Weekly proportion	All‐influenza	Season‐specific
	.1188	Yearly proportion	Subtype	All‐season
	.1189	Rate	Subtype	All‐season
	.1190	Weekly proportion	All‐influenza	All‐season
	.1194	Yearly proportion	All‐influenza	Season‐specific
	.1194	Rate	All‐influenza	Season‐specific
	.1212	Yearly proportion	All‐influenza	All‐season
	.1218	Rate	All‐influenza	All‐season
<65 years	.0943	Weekly proportion	Subtype	All‐season
	.0962	Yearly proportion	Subtype	All‐season
	.0963	Rate	Subtype	All‐season
	.0966	Weekly proportion	All‐influenza	All‐season
	.0986	Rate	All‐influenza	All‐season
	.0991	Yearly proportion	All‐influenza	All‐season
	.0999	Weekly proportion	Subtype	Season‐specific
	.1002	Yearly proportion	Subtype	Season‐specific
	.1002	Rate	Subtype	Season‐specific
	.1013	Weekly proportion	All‐influenza	Season‐specific
	.1013	Yearly proportion	All‐influenza	Season‐specific
	.1013	Rate	All‐influenza	Season‐specific
≥65 years	.8891	Weekly proportion	Subtype	All‐season
	.8902	Rate	Subtype	All‐season
	.8906	Yearly proportion	Subtype	All‐season
	.8953	Weekly proportion	All‐influenza	All‐season
	.8979	Yearly proportion	All‐influenza	All‐season
	.8987	Rate	All‐influenza	All‐season
	.9005	Weekly proportion	Subtype	Season‐specific
	.9015	Yearly proportion	Subtype	Season‐specific
	.9015	Rate	Subtype	Season‐specific
	.9055	Yearly proportion	All‐influenza	Season‐specific
	.9055	Rate	All‐influenza	Season‐specific
	.9057	Weekly proportion	All‐influenza	Season‐specific

a RMSE values are ordered from top to bottom in each age group, with smallest value (best fit) at the top.

### 3.4 Influenza‐associated deaths

Models that use either yearly proportion or rate of season‐specific influenza (sub)types and all‐influenza proxies gave consistently similar estimates in all 3 age groups. The remaining model formulations, however, gave different mortality estimates. While point estimates were different across the models, the 95% confidence intervals overlapped (Tables 3, 4, 5).

**Table 3:** Summary of predicted respiratory mortality rates per 100 000 population for all ages, for South Africa, by year and proxy definition for models, 2009‐2013

	2009		2010		2011		2012		2013		Annual mean^a^	
	Est	95% CI	Est	95% CI	Est	95% CI	Est	95% CI	Est	95% CI	Est	95% CI
All‐season proxy^b^												
Subtypes^c^												
Weekly^d^	5.85	4.98, 6.72	2.36	1.68, 3.05	4.28	3.52, 5.03	1.58	1.11, 2.06	3.71	3.16, 4.26	3.56	2.89, 4.22
Yearly^e^	5.00	4.06, 5.93	1.42	0.74, 2.09	2.84	2.08, 3.60	1.12	0.65, 1.60	2.51	1.99, 3.03	2.58	1.90, 3.25
Rate^f^	4.73	3.90, 5.55	1.61	0.89, 2.34	3.77	2.84, 4.70	1.37	0.81, 1.94	1.92	1.56, 2.28	2.68	2.00, 3.36
All‐influenza^g^												
Weekly	5.94	5.03, 6.84	4.20	3.56, 4.84	4.81	4.08, 5.55	3.01	2.55, 3.47	3.44	2.91, 3.96	4.28	3.63, 4.93
Yearly	4.58	3.66, 5.49	2.94	2.35, 3.53	3.42	2.73, 4.10	2.17	1.74, 2.61	2.32	1.85, 2.78	3.09	2.47, 3.70
Rate	3.94	3.14, 4.75	3.08	2.45, 3.71	4.08	3.24, 4.91	2.51	2.00, 3.02	1.58	1.26, 1.91	3.04	2.42, 3.66
Season‐specific proxy^h^												
Subtypes												
Weekly	6.77	0.96, 12.58	3.21	0.83 5.59	4.51	1.84, 7.18	5.34	4.42, 6.27	3.47	2.24, 4.71	4.66	2.03, 7.30
Yearly	5.96	0.83, 11.09	1.79	−0.64, 4.22	4.35	1.71, 6.99	4.51	3.56, 5.46	3.25	1.93, 4.57	3.97	1.54, 6.41
Rate	5.96	0.82, 11.10	1.79	−0.64, 4.23	4.35	1.71, 6.99	4.50	3.55, 5.45	3.25	1.93, 4.57	3.97	1.35, 6.59
All‐influenza												
Weekly	7.04	5.73, 8.35	1.79	0.62, 2.96	5.22	3.84, 6.60	2.84	1.61, 4.07	3.59	2.01, 5.17	4.10	2.63, 5.56
Yearly	5.29	4.06, 6.51	−0.54	−1.73, 0.64	4.01	2.68, 5.34	1.95	0.73, 3.17	3.75	2.20, 5.29	2.89	1.59, 4.36
Rate	5.29	4.07, 6.52	−0.54	−1.72, 0.64	4.03	2.70, 5.36	1.96	0.74, 3.18	3.74	2.20, 5.28	2.90	1.21, 4.58

Est, point estimate; CI, confidence interval.

a Annual mean mortality rate is calculated by averaging aggregated annual total mortality rates over 5 y.

b Influenza proxy for study period is analyzed as a whole.

c Annual mortality rate obtained by aggregating mortality rate contributed by individual influenza types and subtypes, except when mortality estimate is negative in which case, it is considered as zero for that year in the calculation as negative estimates are biologically non‐meaningful.

d Weekly proportion = number of positive specimens in week/total number of specimens tested in week.

e Yearly proportion = number of positive specimens in week/total number of specimens tested in year.

f Rate = number of positive specimens in week/population in week.

g Proxy consisting of influenza types and subtypes analyzed as individual covariates—A(H1N1)pdm09, A(H3N2), and B.

h Influenza proxy for each season analyzed as individual covariates.

**Table 4:** Summary of predicted respiratory mortality rates per 100 000 population for <65 y, for South Africa, by year and proxy definition for models, 2009‐2013

	2009		2010		2011		2012		2013		Annual mean^a^	
	Est	95% CI	Est	95% CI	Est	95% CI	Est	95% CI	Est	95% CI	Est	95% CI
All‐season proxy^b^												
Subtypes^c^												
Weekly^d^	4.46	3.76, 5.17	1.94	1.38, 2.49	3.53	2.92, 4.14	1.19	0.81, 1.57	3.06	2.62, 3.50	2.84	2.30, 3.37
Yearly^e^	3.71	2.95, 4.47	1.01	0.46, 1.56	2.23	1.61, 2.84	0.68	0.30, 1.07	2.04	1.62, 2.46	1.93	1.39, 2.48
Rate^f^	3.50	2.83, 4.17	1.15	0.56, 1.74	2.95	2.19, 3.70	0.83	0.37, 1.29	1.55	1.26, 1.85	2.00	1.44, 2.55
All‐influenza^g^												
Weekly	4.71	3.97, 5.44	3.33	2.81, 3.85	3.81	3.22, 4.41	2.39	2.02, 2.76	2.72	2.30, 3.15	3.39	2.86, 3.92
Yearly	3.63	2.89, 4.37	2.33	1.85, 2.81	2.71	2.15, 3.27	1.72	1.37, 2.07	1.84	1.46, 2.22	2.45	1.95, 2.95
Rate	3.09	2.43, 3.74	2.41	1.90, 2.93	3.19	2.51, 3.87	1.96	1.55, 2.38	1.24	0.97, 1.50	2.38	1.87, 2.88
Season‐specific proxy^h^												
Subtypes												
Weekly	2.14	1.33, 2.94	1.97	−0.03, 3.98	1.00	−1.25, 3.25	1.32	0.55, 2.10	0.74	−0.27, 1.76	1.44	−0.03, 2.91
Yearly	1.88	1.15, 2.61	1.72	−0.29, 3.73	1.02	−1.16, 3.21	1.23	0.45, 2.01	0.76	−0.31, 1.83	1.32	−0.17, 2.82
Rate	1.88	1.15, 2.62	1.72	−0.29, 3.73	1.02	−1.17, 3.21	1.23	0.44, 2.01	0.76	−0.31, 1.83	1.32	−0.19, 2.83
All‐influenza												
Weekly	2.19	1.20, 3.18	0.44	−0.49, 1.36	1.06	−0.03, 2.14	0.28	−0.67, 1.23	0.59	−0.55, 1.73	0.91	−0.20, 2.02
Yearly	2.04	1.11, 2.97	−0.17	−1.09, 0.75	0.98	−0.06, 2.02	0.20	−0.73, 1.14	0.68	−0.43, 1.79	0.75	−0.36, 1.86
Rate	2.04	1.11, 2.97	−0.17	−1.09, 0.75	0.98	−0.06, 2.02	0.21	−0.73, 1.14	0.68	−0.43, 1.79	0.75	−0.50, 1.99

Est, point estimate; CI, confidence interval.

a Annual mean mortality rate is calculated by averaging aggregated annual total mortality rates over 5 y.

b Influenza proxy for study period is analyzed as a whole.

c Annual mortality rate obtained by aggregating mortality rate contributed by individual influenza types and subtypes, except when mortality estimate is negative in which case, it is considered as zero for that year in the calculation as negative estimates are biologically non‐meaningful.

d Weekly proportion = number of positive specimens in week/total number of specimens tested in week.

e Yearly proportion = number of positive specimens in week/total number of specimens tested in year.

f Rate = number of positive specimens in week/population in week.

g Proxy consisting of influenza types and subtypes analyzed as individual covariates—A(H1N1)pdm09, A(H3N2), and B.

h Influenza proxy for each season analyzed as individual covariates.

**Table 5:** Summary of predicted respiratory mortality rates per 100 000 population for ≥65 y, for South Africa, by year and proxy definition for models, 2009‐2013

	2009		2010		2011		2012		2013		Annual mean^a^	
	Est	95% CI	Est	95% CI	Est	95% CI	Est	95% CI	Est	95% CI	Est	95% CI
All‐season proxy^b^												
Subtypes^c^												
Weekly^d^	31.64	25.01, 38.27	10.41	5.19, 15.63	18.26	12.49, 24.03	9.02	5.42, 12.61	15.84	11.67, 20.02	17.03	11.96, 22.11
Yearly^e^	29.01	22.00, 36.02	8.98	3.93, 14.03	14.30	8.59, 20.00	9.33	5.75, 12.90	11.31	7.42, 15.20	14.59	9.54, 19.63
Rate^f^	27.52	21.33, 33.72	10.29	4.85, 15.72	19.00	12.02, 25.98	11.59	7.35, 15.83	8.69	5.97, 11.42	15.42	10.30, 20.54
All‐influenza^g^												
Weekly	28.97	22.17, 35.77	20.50	15.69, 25.31	23.48	17.97, 28.99	14.70	11.25, 18.15	16.76	12.83, 20.70	20.88	15.98, 25.78
Yearly	22.32	15.54, 29.10	14.35	9.99, 18.70	16.67	11.61, 21.73	10.59	7.37, 13.80	11.30	7.87, 14.74	15.05	10.48, 19.61
Rate	20.13	14.18, 26.08	15.74	11.09, 20.39	20.81	14.66, 26.96	12.81	9.03, 16.60	8.08	5.69, 10.46	15.51	10.93, 20.10
Season‐specific proxy^h^												
Subtypes												
Weekly	11.58	4.33, 18.84	5.01	−2.32, 12.33	9.38	−4.85, 23.61	17.05	10.05, 24.05	7.42	−1.70, 16.54	10.09	0.50, 19.67
Yearly	11.81	5.21, 18.41	4.76	−3.25, 12.77	10.14	−3.81, 24.10	15.43	8.39, 22.48	6.10	−3.50, 15.70	9.65	−0.28, 19.58
Rate	11.81	5.21, 18.41	4.76	−3.26, 12.76	10.13	−3.83, 24.10	15.39	8.36, 22.43	6.08	−3.53, 15.69	9.63	−1.22, 20.49
All‐flu												
Weekly	9.63	0.80, 18.47	1.13	−7.13, 9.39	8.07	−1.60, 17.74	6.96	−1.55, 15.46	7.20	−2.99, 17.38	6.60	−3.33, 16.52
Yearly	7.89	−0.40, 16.18	0.13	−8.13, 8.39	8.20	−1.09, 17.48	6.26	−2.10, 14.63	6.74	−3.18, 16.66	5.84	−4.08, 15.77
Rate	7.90	−0.40, 16.20	0.13	−8.12, 8.39	8.22	−1.07, 17.50	6.28	−2.09, 14.65	6.74	−3.18, 16.66	5.85	−5.29, 17.00

Est, point estimate; CI, confidence interval.

a Annual mean mortality rate is calculated by averaging aggregated annual total mortality rates over 5 y.

b Influenza proxy for study period is analyzed as a whole.

c Annual mortality rate obtained by aggregating mortality rate contributed by individual influenza types and subtypes, except when mortality estimate is negative in which case, it is considered as zero for that year in the calculation as negative estimates are biologically non‐meaningful.

d Weekly proportion = number of positive specimens in week/total number of specimens tested in week.

e Yearly proportion = number of positive specimens in week/total number of specimens tested in year.

f Rate = number of positive specimens in week/population in week.

g Proxy consisting of influenza types and subtypes analyzed as individual covariates—A(H1N1)pdm09, A(H3N2), and B.

h Influenza proxy for each season analyzed as individual covariates.

In the all‐age analysis, the annual mean mortality estimates ranged from 2.58 to 4.66 per 100 000 population. Confidence intervals of the annual mean mortality estimates overlapped with the lowest and highest limit at 1.21‐7.30 per 100 000 population, respectively (Table 3). Our best‐fit model gave the highest annual mean mortality estimate of 4.66 per 100 000 population (95% CI: 2.03, 7.30).

In the 2 age‐stratified groups, the range of annual mean estimates was wider, with the ≥65 years age group experiencing much higher mortality rates. Confidence intervals of annual mean mortality for these 2 age groups also overlapped and included 0. For the <65 years age group, annual mean mortality estimates ranged from 0.75 to 3.39 per 100 000 population and the lowest and highest confidence limits were −0.50 and 3.92, respectively (Table 4). For the ≥65 years age group, the annual mean mortality estimates ranged from 5.84 to 20.88 per 100 000 population and the lowest and highest confidence limits were −5.29 and 25.78, respectively (Table 5). Our best‐fit model gave the highest annual mean mortality estimate of 2.84 per 100 000 population (95% CI: 2.30, 3.37) and 17.03 per 100 000 population (95% CI: 11.96, 22.11) for the <65 and ≥65 years age groups, respectively.

Excess deaths by (sub)type are shown in Figure S2. A direct relationship between dominant influenza (sub)type in circulation with excess mortality in the year was observed with the exception in 2012 where excess deaths were observed to be due to influenza A(H3N2) even though dominant strain in circulation that year was influenza B.

When examining mortality estimates contributed by each influenza (sub)type proxy across all models and age groups, point estimates differed across models, but their 95% confidence intervals generally overlapped. Influenza B gave more negative point estimates compared with influenza A subtypes (Tables S1‐S6).

## 4 DISCUSSION

Using South African SARI surveillance data, our study explored alternative influenza‐incidence proxies and their effect on model fit and respiratory mortality estimates. We tested model formulations considering 3 types of variation in proxy definition: weekly or yearly proportions and population rates; (sub)type‐specific and all‐influenza; and season‐specific or all‐season definitions. Based on model fit, we found that weekly proportion of positive influenza results provided a better fit compared with yearly proportions or rates. Across all age‐group categories, (sub)type‐specific model formulations produced better model fit than all‐influenza proxies. In terms of using season‐specific or all‐season proxies, we got inconsistent results. Season‐specific proxies provided the best model fit for the all‐age model while all‐season proxies provided the best model fit for the age‐specific models. Among all the best‐fitting model formulations, the weekly proportion proxy combined with (sub)type‐specific and either season‐specific and all‐season proxies produced reasonably higher model fit. This would suggest that the more important choice may be whether to use type or season‐specific proxies rather than whether to use a proportion or a rate. Regardless of proxy formulation, none of the point estimates were statistically significantly different from each other for a given age category.

We did not evaluate a proxy of absolute counts of influenza‐positive samples. Assuming testing practices were constant over time, counts should be a reasonable proxy. The population rates we used should be almost equivalent to counts except they adjusted for changes in total underlying population at risk. However, this did not account for changes in surveillance practice or geographic coverage, which were known to occur during the study period. Surprisingly, though, estimates from the population rate proxy did not differ substantially from the yearly proportion proxy. If the number of specimens tested was available, a preferred method may then be to use the proportion positive as a weekly or yearly proportion, which inherently would adjust for the impact of testing and health‐seeking behavior^12^ and would be independent of population size. On the other hand, proportions are sensitive to the size of the denominator which for influenza tests may vary according to the incidence of other pathogens circulating that cause influenza‐like illness. Small numbers of tests can also create problems. For example, sentinel surveillance systems with small numbers of participating sites could have smaller denominators. One positive specimen of 2 specimens tested at the start, or outside, of the influenza season produces a proportion of 50%, even though there is low influenza activity. In previous South African studies, laboratory surveillance data were provided monthly and proxies were calculated as yearly proportions to adjust for possible bias such as different laboratory methods and different specimen sampling over time due to sampling behavior as well as varying changes in total annual number of samples tested.^11^, ^15^, ^16^ In our study, the surveillance data had adequate numbers and it was done on a weekly basis.

We tried season‐ and (sub)type‐specific viral proxies to adjust for any potential year‐to‐year differences between the level of influenza activity and the resulting disease burden.^14^, ^23^ Such differences could be due to variation in virulence arising from antigenic drift of influenza strains,^24^ but also differences in population susceptibility or vaccination coverage and effectiveness over the years. Much wider confidence intervals resulted from season‐specific proxy compared with an all‐season proxy. This could be attributable to reduced statistical power arising from 52 nonzero observations for each season‐specific proxy compared with 260 observations for the all‐season proxy.

It is difficult to compare our estimates with other studies in South Africa as they cover different years (mostly earlier years up to 2009), different modeling methods, different datasets, age groups, and mortality outcomes. Moreover, our study period examined the effects of the 2009 pandemic A(H1N1) strain as the new seasonal H1 strain, which differs from pre‐2009 years, and therefore, mortality effects can be different.^25^ However, in an individual‐level cohort study that looked at influenza‐associated respiratory deaths in the Soweto area in South Africa, their mean estimate of 4.7 (95% CI: 4.1, 5.3) and 8.2 (95% CI: 5.2, 12.5) per 100 000 population for all ages and ≥65 years, respectively,^26^ compares well with our estimate of 4.66 (95% CI: 2.20, 7.12) and 10.09 (95% CI: −2.58, 22.75) per 100 000 population from our model using weekly proportion of season‐specific influenza subtype proxies, assuming that estimates from this site are representative of the country.

So what proxies should be used in practice? We only evaluated proxies with 1 data source in 1 setting. Nevertheless, our findings can be used to provide guidance in other scenarios or to analyze other influenza disease burden outcomes such as hospitalization and intensive care admissions. In this case, our study could inform how influenza proxies should be formulated. We also stress that there is a need to understand how disease burden outcomes are identified and recorded by surveillance systems in order to appropriately adjust for any biases. We have summarized the advantages and disadvantages of using the various influenza proxy formulations in Table 6.

**Table 6:** Summary of advantages and disadvantages of different influenza proxies used in models

Proxy	Advantages	Disadvantages
Weekly proportion	Adjusts for week‐to‐week changes in testing behavior or surveillance coverage.	Can be unstable if denominator is a small count. Does not vary if infection incidence increases but proportion positive is the same. Only possible if number of tests is available.
Yearly proportion	Adjusts for year‐to‐year changes in surveillance practice or coverage. Less subject to small denominator problems than weekly proportion.	Does not adjust for within‐year changes in testing behavior. Does not vary if infection incidence increases for all weeks but proportion positive is the same. Requires whole years of surveillance data. Only possible if number of tests is available.
Weekly rate	Adjusts for population changes. Reflects absolute incidence of infection, if testing behavior or surveillance coverage unchanged.	Does not adjust for testing behavior or surveillance coverage over time
Influenza (sub)types	Higher resolution according to differing epidemiology and virulence of various (sub)types. May provide information for assessing vaccine effectiveness by component strains.	Reduced model parsimony. Reduced statistical power per (sub)type compared with combined influenza series. Proportion of specimens typed or subtyped can vary over time.
All‐influenza	Summary impact of overall influenza. Does not require (sub)type information. Larger counts may lead to greater statistical power to detect an association with mortality.	Does not provide vaccine strain component information.
Season‐specific	Accounts for year‐to‐year changes in virus virulence, surveillance system coverage, and testing behavior.	Reduced statistical power compared with an all‐season proxy.
All‐season	Higher statistical power to detect an association between influenza and mortality compared with season‐specific variables.	Does not account for year‐to‐year changes in virus virulence, surveillance system coverage, and testing.

This study had several limitations. Our study period since the introduction of the pandemic strain only consisted of 5 years, which was relatively short. A longer period would be more ideal to fully encapsulate and understand how influenza‐associated mortality would change over the years. The data quality of the SARI surveillance data was fairly robust but 1 criticism of the program was its representativeness.^20^ The SARI surveillance program only covered 4 out of 9 provinces in the country in 6 sentinel sites, and therefore, it was questionable if the data could nationally represent the viral circulation activity in the population. Also, the number of surveillance sites and output from some of the sites changed over the study period. In addition, there may be a sampling bias toward critically ill patients who required hospitalization and it was not known what proportion of the population this group of patients constituted. Its representation of viral circulation in the population could be overestimated. Other proxies such as ILI for non‐severe cases in the population could be incorporated into the model to balance out the data bias for influenza viral activity. In addition, there have been other studies that explored the interaction of ILI and laboratory surveillance data as a more representative proxy for influenza incidence in the population.^10^ Besides influenza, there was evidence that other viruses such as respiratory syncytial virus (RSV), parainfluenza, and noroviruses may cocirculate with influenza and contributed to excess winter mortality in the elderly,^27^ although previous studies had shown that RSV and influenza did not cocirculate in South Africa at the same time.^15^, ^16^ Nevertheless, our influenza‐associated mortality estimates could have been influenced by other confounding pathogens.

In conclusion, our study provides guidance on choosing appropriate influenza‐incidence proxies for use in estimating the mortality burden of influenza. Improved model fit through use of (sub)type‐specific proxies suggests that the different mortality risk associated with each of influenza A(H1N1)pdm09, A(H3N2), and B appears to be an important factor in estimating mortality. However, varying mortality risk from season to season among influenza strains does not appear to be a clear factor from our study. Regardless of proxy chosen, estimates produced may be broadly consistent and not statistically significantly different. Thus, the large mortality health burden attributable to influenza will still be evident.
