# Supplementary material for: Influenza‐associated mortality in South Africa, 2009‐2013: The importance of choices related to influenza infection proxies
Source: Influenza Other Respir Viruses. 2017 Dec 2;12(1):54–64. doi: 10.1111/irv.12498 (PMC5818357; doi:10.1111/irv.12498)
Supplement: Supplementary file 1 [file IRV-12-54-s001.docx]

**Supporting Information**

| Figure S1 | Time series of SARI influenza surveillance in South Africa, 2009-2013 |
| --- | --- |
| Figure S2 | Time series graph of predicted respiratory mortality from model using weekly-calculated proportion of season-specific influenza subtype proxies showing excess deaths contributed by various influenza (sub)types, referenced against time series of SARI surveillance of Weekly proportion of influenza (sub)types positive samples in South Africa, 2009-2013 |
| Table S1 | Summary of predicted respiratory mortality rates per 100,000 population (95% CI) showing contributions from influenza (sub)types for <65 years for South Africa by proxy definition for models, (sub)type and year for model using all-season influenza (sub)types proxy, 2009-2013 |
| Table S2 | Summary of predicted respiratory mortality rates per 100,000 population (95% CI) showing contributions from influenza (sub)types for ≥65 Years for South Africa by proxy definition for models, (sub)type and year for model using all-season influenza (sub)types proxy, 2009-2013 |
| Table S3 | Summary of predicted respiratory mortality rates per 100,000 population (95% CI) showing contributions from influenza (sub)types for all-ages for South Africa by proxy definition for models, (sub)type and year for model using all-season influenza (sub)types proxy, 2009-2013 |
| Table S4 | Summary of predicted respiratory mortality rates per 100,000 population (95% CI) showing contributions from influenza (sub)types for <65 years for South Africa by proxy definition for models, (sub)type and year for model using season-specific influenza (sub)types proxy, 2009-2013 |
| Table S5 | Summary of predicted respiratory mortality rates per 100,000 population (95% CI) showing contributions from influenza (sub)types for ≥65 years for South Africa by proxy definition for models, (sub)type and year for model using season-specific influenza (sub)types proxy, 2009-2013 |
| Table S6 | Summary of predicted respiratory mortality rates per 100,000 population (95% CI) showing contributions from influenza (sub)types for all-ages for South Africa by proxy definition for models, (sub)type and year for model using season-specific influenza (sub)types proxy, 2009-2013 |

Figure S1. Time series of SARI influenza surveillance in South Africa, 2009-2013. A) Weekly proportion of positive samples; B) Yearly proportion of positive samples; C) Weekly rate of positive samples per 100,000 population.

| A | 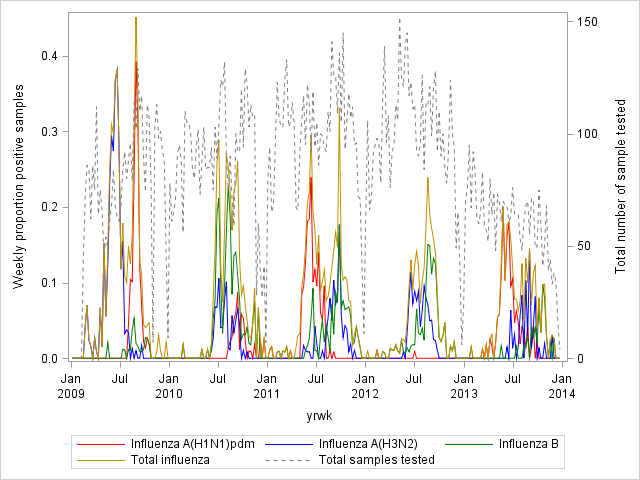 |
| --- | --- |
|  |  |
| B | 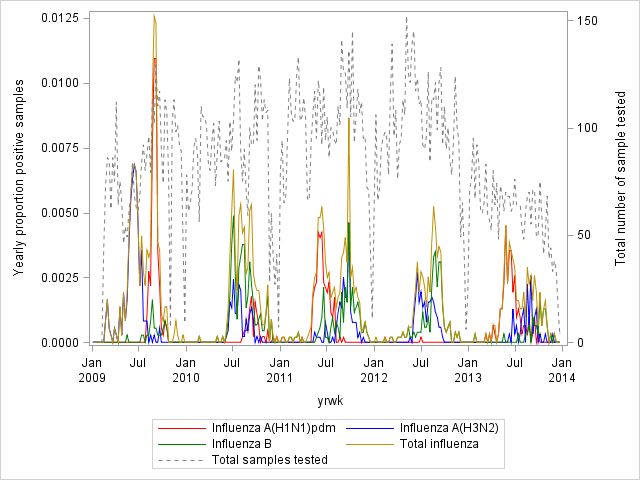 |
|  |  |
| C | 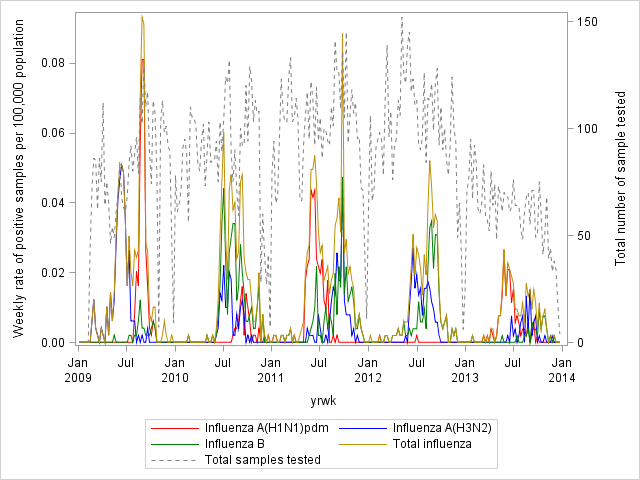 |

Figure S2. Time series graph of predicted respiratory mortality from model using weekly-calculated proportion of season-specific influenza subtype proxies showing excess deaths contributed by various influenza (sub)types, referenced against time series of SARI surveillance of Weekly proportion of influenza (sub)types positive samples in South Africa, 2009-2013. A) <65 years; B) ≥65 years; C) all ages (evaluated as a separate model); D) SARI surveillance of Weekly proportion positive samples.

| A) | 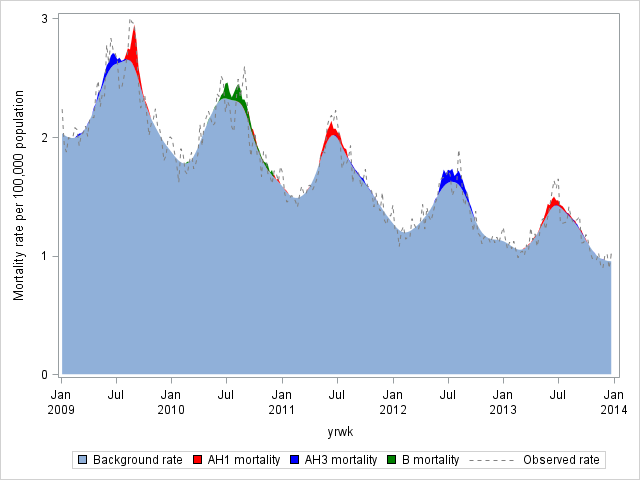 |
| --- | --- |
|  |  |
| B) | 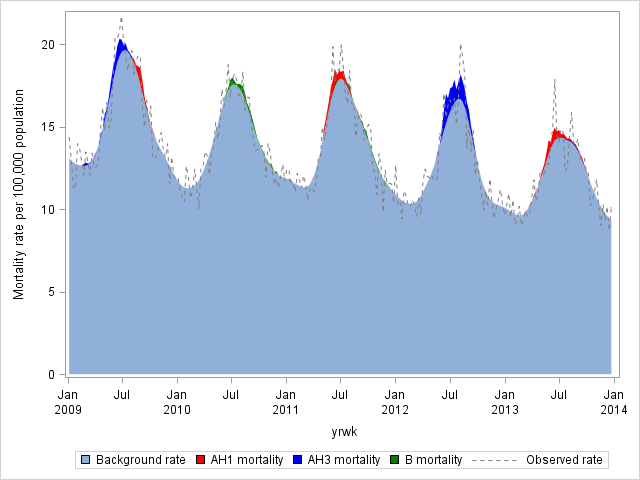 |
|  |  |
| C) | 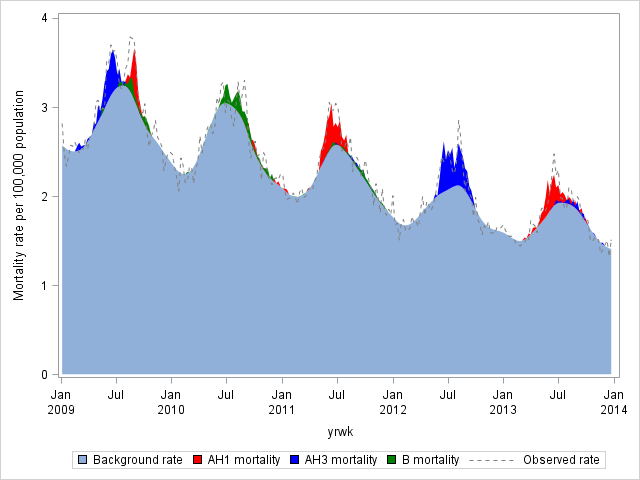 |
|  |  |
| D) | 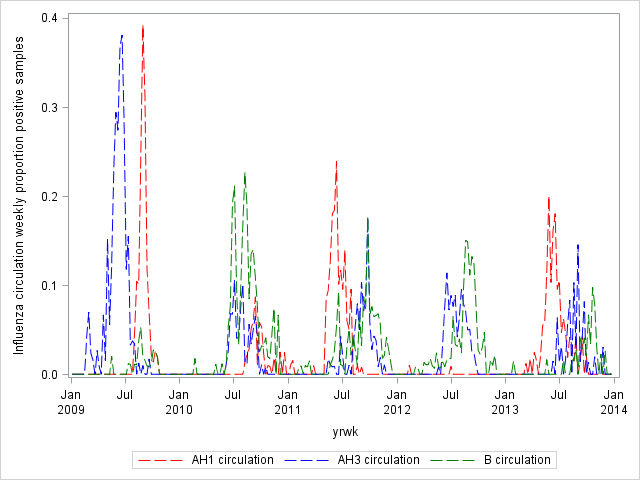 |
|  | 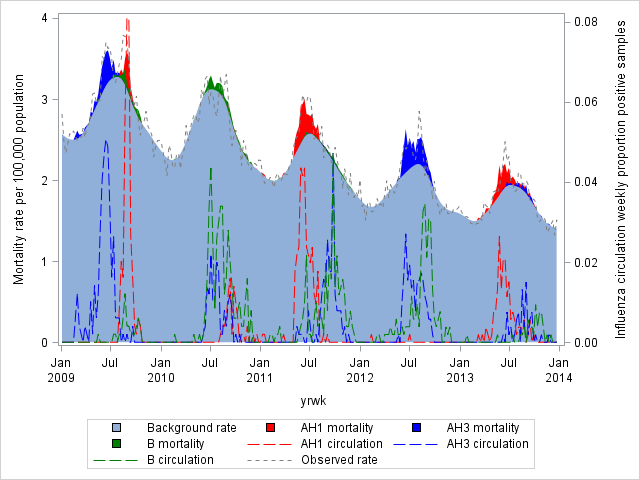 |

Table S1. Summary of predicted respiratory mortality rates per 100,000 population (95% CI) showing contributions from influenza (sub)types for <65 years for South Africa by proxy definition for models, (sub)type and year for model using all-season influenza (sub)types proxy, 2009-2013

|  | **Mortality rate per 100,000 population (95% CI)**  **from model using all-season influenza (sub)types proxy** | | |
| --- | --- | --- | --- |
|  | **Weekly proportion^a^** | **Yearly proportion^b^** | **Rate^c^** |
| **Predicted mortality rate contributed by influenza subtype A(H1N1)pdm09** |  |  |  |
| **2009** | 2.21 (1.85, 2.57) | 2.15 (1.74, 2.57) | 2.10 (1.71, 2.50) |
| **2010** | 0.74 (0.62, 0.87) | 0.48 (0.39, 0.58) | 0.57 (0.47, 0.68) |
| **2011** | 2.45 (2.05, 2.84) | 1.59 (1.28, 1.90) | 2.16 (1.75, 2.57) |
| **2012** | 0.03 (0.02, 0.03) | 0.02 (0.02, 0.02) | 0.03 (0.02, 0.03) |
| **2013** | 2.33 (1.96, 2.71) | 1.62 (1.31, 1.94) | 1.26 (1.02, 1.50) |
| **Predicted mortality rate contributed by influenza subtype A(H3N2)** |  |  |  |
| **2009** | 2.18 (1.52, 2.85) | 1.56 (0.89, 2.22) | 1.40 (0.77, 2.03) |
| **2010** | 0.64 (0.44, 0.83) | 0.53 (0.30, 0.75) | 0.57 (0.32, 0.83) |
| **2011** | 0.74 (0.52, 0.97) | 0.64 (0.36, 0.91) | 0.79 (0.43, 1.15) |
| **2012** | 0.82 (0.57, 1.07) | 0.66 (0.38, 0.95) | 0.80 (0.44, 1.16) |
| **2013** | 0.58 (0.40, 0.76) | 0.41 (0.24, 0.59) | 0.29 (0.16, 0.43) |
| **Predicted mortality rate contributed by influenza type B** |  |  |  |
| **2009** | 0.07 (-0.02, 0.16) | -0.04 (-0.15, 0.08) | -0.04 (-0.13, 0.05) |
| **2010** | 0.56 (-0.15, 1.27) | -0.22 (-0.93, 0.49) | -0.31 (-1.01, 0.39) |
| **2011** | 0.34 (-0.10, 0.78) | -0.15 (-0.61, 0.32) | -0.23 (-0.75, 0.29) |
| **2012** | 0.34 (-0.09, 0.77) | -0.14 (-0.59, 0.31) | -0.21 (-0.7, 0.27) |
| **2013** | 0.14 (-0.04, 0.33) | -0.05 (-0.22, 0.11) | -0.05 (-0.15, 0.06) |
| **Total predicted mortality rate^d^** |  |  |  |
| **2009** | 4.46 (3.76, 5.17) | 3.71 (2.95, 4.47) | 3.50 (2.83, 4.17) |
| **2010** | 1.94 (1.38, 2.49) | 1.01 (0.46, 1.56) | 1.15 (0.56, 1.74) |
| **2011** | 3.53 (2.92, 4.14) | 2.23 (1.61, 2.84) | 2.95 (2.19, 3.70) |
| **2012** | 1.19 (0.81, 1.57) | 0.68 (0.30, 1.07) | 0.83 (0.37, 1.29) |
| **2013** | 3.06 (2.62, 3.50) | 2.04 (1.62, 2.46) | 1.55 (1.26, 1.85) |
| **Annual mean^e^** | 2.84 (2.30, 3.37) | 1.93 (1.39, 2.48) | 2.00 (1.44, 2.55) |

^a^ Weekly proportion = number of positive specimens in week / total number of specimens tested in week

^b^ Yearly proportion = number of positive specimens in week / total number of specimens tested in year
^c^ Rate = number of positive specimens in week / population in week

^d^ Annual mortality rate obtained by aggregating mortality rate contributed by individual influenza types and subtypes, except when mortality estimate is negative in which case, it is considered as zero for that year in the calculation since negative estimates are biologically non-meaningful.

^e^ Annual mean mortality rate is calculated by averaging aggregated annual total mortality rates over 5 years.

Table S2. Summary of predicted respiratory mortality rates per 100,000 population (95% CI) showing contributions from influenza (sub)types for ≥65 Years for South Africa by proxy definition for models, (sub)type and year for model using all-season influenza (sub)types proxy, 2009-2013

|  | **Mortality rate per 100,000 population (95% CI)**  **from model using all-season influenza (sub)types proxy** | | |
| --- | --- | --- | --- |
|  | **Weekly proportion^a^** | **Yearly proportion^b^** | **Rate^c^** |
| **Predicted mortality rate contributed by influenza subtype A(H1N1)pdm09** |  |  |  |
| **2009** | 9.17 (5.79, 12.56) | 7.35 (3.48, 11.21) | 7.46 (3.79, 11.13) |
| **2010** | 3.09 (1.95, 4.23) | 1.65 (0.78, 2.52) | 2.04 (1.03, 3.04) |
| **2011** | 10.14 (6.40, 13.88) | 5.42 (2.57, 8.28) | 7.66 (3.89, 11.43) |
| **2012** | 0.12 (0.08, 0.16) | 0.07 (0.03, 0.10) | 0.09 (0.05, 0.13) |
| **2013** | 9.68 (6.11, 13.25) | 5.54 (2.62, 8.45) | 4.47 (2.27, 6.67) |
| **Predicted mortality rate contributed by influenza subtype A(H3N2)** |  |  |  |
| **2009** | 22.36 (16.09, 28.64) | 21.66 (15.49, 27.84) | 20.06 (14.23, 25.90) |
| **2010** | 6.53 (4.70, 8.36) | 7.33 (5.24, 9.42) | 8.25 (5.85, 10.65) |
| **2011** | 7.63 (5.49, 9.77) | 8.87 (6.34, 11.40) | 11.34 (8.04, 14.63) |
| **2012** | 8.41 (6.05, 10.77) | 9.26 (6.62, 11.90) | 11.50 (8.16, 14.85) |
| **2013** | 5.96 (4.28, 7.63) | 5.77 (4.13, 7.42) | 4.22 (2.99, 5.45) |
| **Predicted mortality rate contributed by influenza type B** |  |  |  |
| **2009** | 0.10 (-0.75, 0.95) | -0.22 (-1.29, 0.84) | -0.37 (-1.22, 0.49) |
| **2010** | 0.79 (-5.90, 7.49) | -1.38 (-7.96, 5.20) | -2.76 (-9.24, 3.73) |
| **2011** | 0.49 (-3.63, 4.61) | -0.90 (-5.20, 3.39) | -2.04 (-6.85, 2.76) |
| **2012** | 0.48 (-3.61, 4.58) | -0.88 (-5.04, 3.29) | -1.92 (-6.45, 2.60) |
| **2013** | 0.20 (-1.52, 1.93) | -0.32 (-1.83, 1.20) | -0.41 (-1.38, 0.56) |
| **Total predicted mortality rate^d^** |  |  |  |
| **2009** | 31.64 (25.01, 38.27) | 29.01 (22.00, 36.02) | 27.52 (21.33, 33.72) |
| **2010** | 10.41 (5.19, 15.63) | 8.98 (3.93, 14.03) | 10.29 (4.85, 15.72) |
| **2011** | 18.26 (12.49, 24.03) | 14.30 (8.59, 20.00) | 19.00 (12.02, 25.98) |
| **2012** | 9.02 (5.42, 12.61) | 9.33 (5.75, 12.90) | 11.59 (7.35, 15.83) |
| **2013** | 15.84 (11.67, 20.02) | 11.31 (7.42, 15.20) | 8.69 (5.97, 11.42) |
| **Annual mean^e^** | 17.03 (11.96, 22.11) | 14.58 (9.54, 19.63) | 15.42 (10.30, 20.54) |

^a^ Weekly proportion = number of positive specimens in week / total number of specimens tested in week

^b^ Yearly proportion = number of positive specimens in week / total number of specimens tested in year
^c^ Rate = number of positive specimens in week / population in week

^d^ Annual mortality rate obtained by aggregating mortality rate contributed by individual influenza types and subtypes, except when mortality estimate is negative in which case, it is considered as zero for that year in the calculation since negative estimates are biologically non-meaningful.

^e^ Annual mean mortality rate is calculated by averaging aggregated annual total mortality rates over 5 years.

Table S3. Summary of predicted respiratory mortality rates per 100,000 population (95% CI) showing contributions from influenza (sub)types for all-ages for South Africa by proxy definition for models, (sub)type and year for model using all-season influenza (sub)types proxy, 2009-2013

|  | **Mortality rate per 100,000 population (95% CI)**  **from model using all-season influenza (sub)types proxy** | | |
| --- | --- | --- | --- |
|  | **Weekly proportion^a^** | **Yearly proportion^b^** | **Rate^c^** |
| **Predicted mortality rate contributed by influenza subtype A(H1N1)pdm09** |  |  |  |
| **2009** | 2.57 (2.12, 3.01) | 2.42 (1.90, 2.94) | 2.38 (1.89, 2.87) |
| **2010** | 0.86 (0.71, 1.01) | 0.54 (0.43, 0.66) | 0.65 (0.52, 0.78) |
| **2011** | 2.84 (2.35, 3.33) | 1.79 (1.41, 2.17) | 2.44 (1.94, 2.95) |
| **2012** | 0.03 (0.03, 0.04) | 0.02 (0.02, 0.03) | 0.03 (0.02, 0.03) |
| **2013** | 2.71 (2.24, 3.18) | 1.82 (1.44, 2.21) | 1.43 (1.13, 1.72) |
| **Predicted mortality rate contributed by influenza subtype A(H3N2)** |  |  |  |
| **2009** | 3.21 (2.39, 4.03) | 2.58 (1.75, 3.40) | 2.35 (1.57, 3.13) |
| **2010** | 0.94 (0.70, 1.18) | 0.87 (0.59, 1.15) | 0.97 (0.64, 1.29) |
| **2011** | 1.10 (0.81, 1.38) | 1.06 (0.72, 1.39) | 1.33 (0.89, 1.77) |
| **2012** | 1.21 (0.90, 1.52) | 1.10 (0.75, 1.45) | 1.35 (0.90, 1.79) |
| **2013** | 0.85 (0.64, 1.07) | 0.69 (0.47, 0.91) | 0.49 (0.33, 0.66) |
| **Predicted mortality rate contributed by influenza type B** |  |  |  |
| **2009** | 0.07 (-0.04, 0.18) | -0.05 (-0.19, 0.09) | -0.06 (-0.17, 0.05) |
| **2010** | 0.56 (-0.32, 1.44) | -0.30 (-1.17, 0.58) | -0.45 (-1.32, 0.42) |
| **2011** | 0.35 (-0.19, 0.89) | -0.19 (-0.77, 0.38) | -0.33 (-0.98, 0.31) |
| **2012** | 0.34 (-0.19, 0.88) | -0.19 (-0.74, 0.37) | -0.31 (-0.92, 0.29) |
| **2013** | 0.15 (-0.08, 0.37) | -0.07 (-0.27, 0.13) | -0.07 (-0.20, 0.06) |
| **Total predicted mortality rate^d^** |  |  |  |
| **2009** | 5.85 (4.98, 6.72) | 5.00 (4.06, 5.93) | 4.73 (3.90, 5.55) |
| **2010** | 2.36 (1.68, 3.05) | 1.42 (0.74, 2.09) | 1.61 (0.89, 2.34) |
| **2011** | 4.28 (3.52, 5.03) | 2.84 (2.08, 3.60) | 3.77 (2.84, 4.70) |
| **2012** | 1.58 (1.11, 2.06) | 1.12 (0.65, 1.60) | 1.37 (0.81, 1.94) |
| **2013** | 3.71 (3.16, 4.26) | 2.51 (1.99, 3.03) | 1.92 (1.56, 2.28) |
| **Annual mean^e^** | 3.56 (2.89, 4.22) | 2.58 (1.90, 3.25) | 2.68 (2.00, 3.36) |

^a^ Weekly proportion = number of positive specimens in week / total number of specimens tested in week

^b^ Yearly proportion = number of positive specimens in week / total number of specimens tested in year
^c^ Rate = number of positive specimens in week / population in week

^d^ Annual mortality rate obtained by aggregating mortality rate contributed by individual influenza types and subtypes, except when mortality estimate is negative in which case, it is considered as zero for that year in the calculation since negative estimates are biologically non-meaningful.

^e^ Annual mean mortality rate is calculated by averaging aggregated annual total mortality rates over 5 years.

Table S4. Summary of predicted respiratory mortality rates per 100,000 population (95% CI) showing contributions from influenza (sub)types for <65 years for South Africa by proxy definition for models, (sub)type and year for model using season-specific influenza (sub)types proxy, 2009-2013

|  | **Mortality rate per 100,000 population (95% CI)**  **from model using season-specific influenza (sub)types proxy** | | |
| --- | --- | --- | --- |
|  | **Weekly proportion^a^** | **Yearly proportion^b^** | **Rate^c^** |
| **Predicted mortality rate contributed by influenza subtype A(H1N1)pdm09** |  |  |  |
| **2009** | 1.35 (0.68, 2.01) | 1.22 (0.54, 1.89) | 1.22 (0.54, 1.90) |
| **2010** | 0.48 (-0.27, 1.23) | 0.19 (-0.51, 0.90) | 0.19 (-0.51, 0.90) |
| **2011** | 0.85 (0.11, 1.60) | 0.90 (0.16, 1.63) | 0.90 (0.16, 1.64) |
| **2012** | -0.05 (-0.35, 0.25) | -0.05 (-0.35, 0.25) | -0.05 (-0.35, 0.25) |
| **2013** | 0.68 (-0.15, 1.50) | 0.67 (-0.14, 1.49) | 0.67 (-0.14, 1.49) |
| **Predicted mortality rate contributed by influenza subtype A(H3N2)** |  |  |  |
| **2009** | 0.79 (0.05, 1.54) | 0.67 (-0.09, 1.42) | 0.67 (-0.09, 1.42) |
| **2010** | -1.38 (-2.64, -0.12) | -1.64 (-2.95, -0.33) | -1.64 (-2.95, -0.33) |
| **2011** | 0.14 (-1.02, 1.30) | 0.11 (-1.08, 1.29) | 0.11 (-1.08, 1.29) |
| **2012** | 1.32 (0.32, 2.32) | 1.23 (0.23, 2.23) | 1.23 (0.22, 2.23) |
| **2013** | 0.07 (-0.62, 0.75) | 0.09 (-0.61, 0.78) | 0.09 (-0.61, 0.78) |
| **Predicted mortality rate contributed by influenza type B** |  |  |  |
| **2009** | -0.13 (-1.03, 0.78) | -0.09 (-1.00, 0.82) | -0.09 (-1.00, 0.82) |
| **2010** | 1.50 (-0.05, 3.04) | 1.53 (-0.16, 3.21) | 1.53 (-0.16, 3.21) |
| **2011** | 0.00 (-1.54, 1.54) | 0.01 (-1.53, 1.56 | 0.02 (-1.53, 1.56) |
| **2012** | -0.98 (-2.03, 0.06) | -0.92 (-1.92, 0.09) | -0.91 (-1.92, 0.09) |
| **2013** | -0.34 (-1.09, 0.40) | -0.30 (-1.07, 0.48) | -0.30 (-1.07, 0.48) |
| **Total predicted mortality rate^d^** |  |  |  |
| **2009** | 2.14 (1.33, 2.94) | 1.88 (1.15, 2.61) | 1.88 (1.15, 2.62) |
| **2010** | 1.97 (-0.03, 3.98) | 1.72 (-0.29, 3.73) | 1.72 (-0.29, 3.73) |
| **2011** | 1.00 (-1.25, 3.25) | 1.02 (-1.16, 3.21) | 1.02 (-1.17, 3.21) |
| **2012** | 1.32 (0.55, 2.10) | 1.23 (0.45, 2.01) | 1.23 (0.44, 2.01) |
| **2013** | 0.74 (-0.27, 1.76) | 0.76 (-0.31, 1.83) | 0.76 (-0.31, 1.83) |
| **Annual mean^e^** | 1.44 (-0.03, 2.91) | 1.32 (-0.17, 2.82) | 1.32 (-0.19, 2.83) |

^a^ Weekly proportion = number of positive specimens in week / total number of specimens tested in week

^b^ Yearly proportion = number of positive specimens in week / total number of specimens tested in year
^c^ Rate = number of positive specimens in week / population in week

^d^ Annual mortality rate obtained by aggregating mortality rate contributed by individual influenza types and subtypes, except when mortality estimate is negative in which case, it is considered as zero for that year in the calculation since negative estimates are biologically non-meaningful.

^e^ Annual mean mortality rate is calculated by averaging aggregated annual total mortality rates over 5 years.

Table S5. Summary of predicted respiratory mortality rates per 100,000 population (95% CI) showing contributions from influenza (sub)types for ≥65 years for South Africa by proxy definition for models, (sub)type and year for model using season-specific influenza (sub)types proxy, 2009-2013

|  | **Mortality rate per 100,000 population (95% CI)**  **from model using season-specific influenza (sub)types proxy** | | |
| --- | --- | --- | --- |
|  | **Weekly proportion^a^** | **Yearly proportion^b^** | **Rate^c^** |
| **Predicted mortality rate contributed by influenza subtype A(H1N1)pdm09** |  |  |  |
| **2009** | 3.66 (-2.36, 9.68) | 2.80 (-3.32, 8.92) | 2.80 (-3.32, 8.92) |
| **2010** | -2.73 (-9.48, 4.03) | -3.27 (-9.60, 3.06) | -3.27 (-9.60, 3.06) |
| **2011** | 7.04 (0.32, 13.75) | 7.21 (0.59, 13.84) | 7.21 (0.58, 13.83) |
| **2012** | -0.68 (-3.36, 2.01) | -0.67 (-3.39, 2.05) | -0.67 (-3.38, 2.05) |
| **2013** | 6.74 (-0.69, 14.16) | 5.63 (-1.73, 12.99) | 5.63 (-1.73, 12.98) |
| **Predicted mortality rate contributed by influenza subtype A(H3N2)** |  |  |  |
| **2009** | 7.92 (1.22, 14.63) | 9.01 (2.23, 15.79) | 9.01 (2.23, 15.79) |
| **2010** | -1.78 (-13.12, 9.56) | -1.95 (-13.76, 9.86) | -1.94 (13.76, 9.88) |
| **2011** | -2.10 (-12.56, 8.37) | -1.63 (-12.29, 9.02) | -1.63 (-12.28, 9.01) |
| **2012** | 17.05 (8.04, 26.05) | 15.43 (6.40, 24.47) | 15.39 (6.37, 24.41) |
| **2013** | 0.69 (-5.47, 6.84) | 0.46 (-5.82, 6.75) | 0.46 (-5.83, 6.74) |
| **Predicted mortality rate contributed by influenza type B** |  |  |  |
| **2009** | -3.55 (-11.72, 4.62) | -2.69 (-10.90, 5.51) | -2.70 (-10.90, 5.51) |
| **2010** | 5.01 (-8.94, 18.95) | 4.76 (-10.41, 19.93) | 4.75 (-10.42, 19.92) |
| **2011** | 2.34 (-11.54, 16.22) | 2.93 (-10.96, 16.81) | 2.93 (-10.95, 16.80) |
| **2012** | -9.92 (-19.34, -0.50) | -8.46 (-17.53, 0.60) | -8.44 (-17.50, 0.63) |
| **2013** | -1.19 (-7.91, 5.53) | -0.05 (-7.02, 6.91) | -0.04 (-7.01, 6.92) |
| **Total predicted mortality rate^d^** |  |  |  |
| **2009** | 11.58 (4.33, 18.84) | 11.81 (5.21, 18.41) | 11.81 (5.21, 18.41) |
| **2010** | 5.01 (-2.32, 12.33) | 4.76 (-3.25, 12.77) | 4.76 (-3.26, 12.76) |
| **2011** | 9.38 (-4.85, 23.61) | 10.14 (-3.81, 24.10) | 10.13 (-3.83, 24.10) |
| **2012** | 17.05 (10.05, 24.05) | 15.43 (8.39, 22.48) | 15.39 (8.36, 22.43) |
| **2013** | 7.42 (-1.70, 16.54) | 6.10 (-3.50, 15.70) | 6.08 (-3.53, 15.69) |
| **Annual mean^e^** | 10.09 (0.50, 19.67) | 9.65 (-0.28, 19.58) | 9.63 (-1.22, 20.49) |

^a^ Weekly proportion = number of positive specimens in week / total number of specimens tested in week

^b^ Yearly proportion = number of positive specimens in week / total number of specimens tested in year
^c^ Rate = number of positive specimens in week / population in week

^d^ Annual mortality rate obtained by aggregating mortality rate contributed by individual influenza types and subtypes, except when mortality estimate is negative in which case, it is considered as zero for that year in the calculation since negative estimates are biologically non-meaningful.

^e^ Annual mean mortality rate is calculated by averaging aggregated annual total mortality rates over 5 years.

Table S6. Summary of predicted respiratory mortality rates per 100,000 population (95% CI) showing contributions from influenza (sub)types for all-ages for South Africa by proxy definition for models, (sub)type and year for model using season-specific influenza (sub)types proxy, 2009-2013

|  | **Mortality rate per 100,000 population (95% CI)**  **from model using season-specific influenza (sub)types proxy** | | |
| --- | --- | --- | --- |
|  | **Weekly proportion^a^** | **Yearly proportion^b^** | **Rate^c^** |
| **Predicted mortality rate contributed by influenza subtype A(H1N1)pdm09** |  |  |  |
| **2009** | 2.16 (1.36, 2.63) | 1.80 (0.98, 2.62) | 1.80 (0.98, 2.62) |
| **2010** | 0.80 (-0.09, 1.69) | 0.13 (-0.71, 0.98) | 0.13 (-0.71, 0.98) |
| **2011** | 3.38 (2.49, 4.27) | 3.54 (2.65, 4.44) | 3.54 (2.65, 4.44) |
| **2012** | -0.07 (-0.43, 0.28) | -0.07 (-0.44, 0.29) | -0.07 (-0.44, 0.29) |
| **2013** | 2.96 (1.93, 4.00) | 2.69 (1.65, 3.73) | 2.69 (1.65, 3.72) |
| **Predicted mortality rate contributed by influenza subtype A(H3N2)** |  |  |  |
| **2009** | 3.56 (2.63, 4.49) | 3.25 (2.29, 4.21) | 3.25 (2.29, 4.21) |
| **2010** | -1.29 (-2.78, 0.20) | -1.89 (-3.46, -0.31) | -1.89 (-3.46, -0.31) |
| **2011** | 0.46 (-0.92, 1.83) | 0.27 (-1.16, 1.69) | 0.27 (-1.15, 1.69) |
| **2012** | 5.34 (4.15, 6.53) | 4.51 (3.30, 5.73) | 4.50 (3.28, 5.71) |
| **2013** | 0.51 (-0.32, 1.34) | 0.56 (-0.30, 1.42) | 0.56 (-0.30, 1.42) |
| **Predicted mortality rate contributed by influenza type B** |  |  |  |
| **2009** | 1.06 (-0.03, 2.15) | 0.91 (-0.21, 2.02) | 0.91 (-0.21, 2.02) |
| **2010** | 2.41 (0.57, 4.25) | 1.66 (-0.38, 3.70) | 1.66 (-0.38, 3.70) |
| **2011** | 0.68 (-1.16, 2.52) | 0.54 (-1.33, 2.41) | 0.54 (-1.33, 2.41) |
| **2012** | -1.11 (-2.37, 0.15) | -1.31 (-2.54, -0.08) | -1.30 (-2.54, -0.07) |
| **2013** | -0.44 (-1.37, 0.49) | -0.21 (-1.19, 0.77) | -0.21 (-1.19, 0.77) |
| **Total predicted mortality rate^d^** |  |  |  |
| **2009** | 6.77 (0.96, 12.58) | 5.96 (0.83, 11.09) | 5.96 (0.82, 11.10) |
| **2010** | 3.21 (0.83, 5.59) | 1.79 (-0.64, 4.22) | 1.79 (-0.64, 4.23) |
| **2011** | 4.51 (1.84, 7.18) | 4.35 (1.71, 6.99) | 4.35 (1.71, 6.99) |
| **2012** | 5.34 (4.42, 6.27) | 4.51 (3.56, 5.46) | 4.50 (3.55, 5.45) |
| **2013** | 3.47 (2.24, 4.71) | 3.25 (1.93, 4.57) | 3.25 (1.93, 4.57) |
| **Annual mean^e^** | 4.66 (2.03, 7.30) | 3.97 (1.54, 6.41) | 3.97 (1.35, 6.59) |

^a^ Weekly proportion = number of positive specimens in week / total number of specimens tested in week

^b^ Yearly proportion = number of positive specimens in week / total number of specimens tested in year
^c^ Rate = number of positive specimens in week / population in week

^d^ Annual mortality rate obtained by aggregating mortality rate contributed by individual influenza types and subtypes, except when mortality estimate is negative in which case, it is considered as zero for that year in the calculation since negative estimates are biologically non-meaningful.

^e^ Annual mean mortality rate is calculated by averaging aggregated annual total mortality rates over 5 years.
